# Comparison of the Bacterial Gut Microbiome of North American *Triatoma* spp. With and Without *Trypanosoma cruzi*

**DOI:** 10.3389/fmicb.2020.00364

**Published:** 2020-03-13

**Authors:** Allison E. Mann, Elizabeth A. Mitchell, Yan Zhang, Rachel Curtis-Robles, Santosh Thapa, Sarah A. Hamer, Michael S. Allen

**Affiliations:** ^1^Tick-Borne Disease Research Laboratory, Department of Microbiology, Immunology, and Genetics, University of North Texas Health Science Center, Fort Worth, TX, United States; ^2^Department of Veterinary Integrative Biosciences, Texas A&M University, College Station, TX, United States; ^3^Texas Children’s Microbiome Center, Texas Children’s Hospital, Houston, TX, United States; ^4^Department of Pathology and Immunology, Baylor College of Medicine, Houston, TX, United States

**Keywords:** *Triatoma* spp., microbiome, Chagas disease, insect-vectored pathogen, *Trypanosoma cruzi*

## Abstract

Chagas disease, caused by the hemoflagellate protist *Trypanosoma cruzi*, affects nearly 6 million people worldwide, mainly in Latin America. Hematophagous triatomine insects (“kissing bugs”) are the primary vectors of *T. cruzi* throughout the Americas and feed on a variety of animals, including humans. Control of triatomines is central to the control of *T. cruzi* infection. Recent advances in mitigation of other insect-borne diseases via the manipulation of insect-associated bacteria as a way to halt or slow disease transmission has opened questions to the applicability of these methods to Chagas disease vectors. Few studies have examined the hindgut microbiome of triatomines found in North America. In the current study, two species of triatomines were collected across Texas, United States, screened for the presence of *T. cruzi*, and analyzed for the bacterial composition of their hindguts using a 16S rRNA gene-fragment metabarcoding approach. We compared diversity of microbial community profiles across 74 triatomine insects to address the hypothesis that the richness and abundance of bacterial groups differ by *T. cruzi* infection and strain type, blood meal engorgement status, insect species, sex, and collection location. The gut microbial community of individual triatomines was characterized by low intraindividual taxonomic diversity and high interindividual variation that was weakly predicted by triatomine species, and was not predicted by triatomine sex, collection location, *T. cruzi* infection status, or blood meal score. However, we did find bacterial groups enriched in *T. cruzi*-positive individuals, including Enterobacterales, and *Petrimonas*. Additionally, we detected *Salmonella enterica* subspecies *diarizonae* in three triatomine individuals; this species is commonly associated with reptiles and domesticated animals and is a pathogen of humans. These data suggest that *Triatoma* spp. in Texas have variable patterns of colonized and transient bacteria, and may aid in development of novel means to interfere with transmission of the Chagas disease parasite *T. cruzi*. Deeper understanding of the effects of parasite infection on diverse insect vector microbiomes may highlight disease transmission risk and facilitate discovery of possible intervention strategies for biological control of this emerging vector-borne disease of global health significance.

## Introduction

Chagas disease is estimated to affect approximately 6 million people across the Americas (World Health Organization [WHO], 2015) and is a significant cause of cardiovascular death in endemic areas (Rassi et al., 2009). Caused by the hemoflagellate protist *Trypanosoma cruzi*, the principal method of transmission is via the infective feces of hematophagous triatomine insect vectors within the family Reduviidae, with a fewer number of cases transmitted congenitally, through blood transfusion, organ transplantation, and direct ingestion (Kjos et al., 2008; Bern et al., 2011; Picado et al., 2018). While the majority of *T. cruzi* infection cases occur in Latin America, there has been a rise in reports of Chagas disease in the United States and Europe (Lescure et al., 2008; Bern and Montgomery, 2009; Jackson et al., 2009; Pérez de Ayala et al., 2009; Bern et al., 2011; Pérez-Ayala et al., 2011). In the United States, eleven triatomine species have been identified. Texas, home to seven *Triatoma* species, is the state with the greatest triatomine species diversity (Bern et al., 2011). *T. cruzi* infection prevalence in triatomines collected in Texas is greater than 50% in some *Triatoma* spp (Kjos et al., 2008; Curtis-Robles et al., 2018a), with discrete typing units (DTU) TcI and TcIV most commonly represented (Roellig et al., 2013; Brenière et al., 2016; Curtis-Robles et al., 2018a). Although triatomines found in the United States are generally considered sylvatic, these vectors are regularly encountered in and around human dwellings (Kjos et al., 2008; Wozniak et al., 2015; Curtis-Robles et al., 2018b). Wildlife and domestic canines are infected with *T. cruzi* throughout Texas and across the southern United States (Beard et al., 2003; Kjos et al., 2008; Brown et al., 2010; Curtis-Robles et al., 2017; Hodo and Hamer, 2017).

The bacterial microbiome living in the gastrointestinal tract or other organs of a host has increasingly been investigated as a potential target for intervention in efforts to limit disease transmission by a variety of arthropod vectors (Cirimotich et al., 2011; Crotti et al., 2012; Geiger et al., 2013; Hegde et al., 2015; Taracena et al., 2015; Vieira et al., 2015; Buarque et al., 2016). To date, few studies have examined the gut microbiome of triatomine vectors, and even fewer have looked at *Triatoma* spp. collected in the United States (da Mota et al., 2012; Gumiel et al., 2015; Díaz et al., 2016; Montoya-Porras et al., 2018; Rodríguez-Ruano et al., 2018; Waltmann et al., 2019). The composition and diversity of insect gut bacteria could play a significant role, not only in the life cycle of the insect itself (Rodríguez-Ruano et al., 2018), but also in relation to the life cycles of the parasites that they vector, including whether the insect is able to competently harbor and/or transmit various microbes to hosts (Engel and Moran, 2013; Goodrich Julia et al., 2014; Teotônio et al., 2019).

Bacterial gut communities among different arthropods are known to vary widely in composition, function, and response to the environment (Engel and Moran, 2013; Menchaca et al., 2013). Gut microbiome studies in South American triatomines further indicate this high level of variability (da Mota et al., 2012; Gumiel et al., 2015; Díaz et al., 2016; Montoya-Porras et al., 2018; Waltmann et al., 2019). da Mota et al. (2012) report relatively low overall species complexity in the gut microbiomes of South American triatomine bugs. Similar findings are reported for two species of North American *Triatoma*, in which the authors further conclude that *T. cruzi* infection leads to apparent increases in bacterial diversity within the triatomine host gut (Rodríguez-Ruano et al., 2018). The finding of increased bacterial diversity in the hindgut of *T. cruzi*-infected triatomines is consistent with previously reported changes in the gut microbiome and host physiology upon *T. cruzi* infection in South American triatomines (Vieira et al., 2015; Díaz et al., 2016), though another study found no impact of *T. cruzi* infection on alpha diversity metrics in both lab-reared and wild triatomine individuals (Waltmann et al., 2019). These studies point to the potential shaping of the triatomine gut microbial population by *T. cruzi* during infection but also the dynamic nature of the hindgut community that may be governed by a variety of factors including interspecies variation, collection location, sex, life cycle stage, and wild versus lab-reared populations. Differences in microbial community response to *T. cruzi* infection among triatomines may also reveal possible alternative control strategies based on the manipulation of the gut microbiome. It is therefore beneficial to explore how gut bacterial colonization of *Triatoma* spp. found in the United States differ in relation to various characteristics associated with different *Triatoma* species and with respect to *T. cruzi* infection status.

To explore these issues, this study used massively parallel sequencing of the V4 region of the 16S rRNA gene to assess the gut bacterial composition of two *Triatoma* spp (*Triatoma gerstaeckeri n* = 55 and *Triatoma sanguisuga n* = 19) collected from across Texas. Isolated bacterial sequences were compared across triatomine individuals with regard to *T. cruzi* infection status, *T. cruzi* DTU classification, blood engorgement status, triatomine species, sex, and geographic location.

## Materials and Methods

### Sample Selection, Preparation, and Testing

Using a stratified random sampling design, triatomines were selected from Texas specimens that had been collected via a citizen science program (Curtis-Robles et al., 2015) and traditional entomological techniques (black light, mercury vapor light, and active searching of environments) (Curtis-Robles et al., 2018b) between June 2013 and September 2014. Individuals were sampled to represent the two most frequently encountered species (*T. gerstaeckeri* and *T. sanguisuga*) in the collection as well as three less-frequently encountered species (*Triatoma indictiva*, *Triatoma lecticularia*, and *Triatoma protracta*), with further stratification by sex (approximately equal numbers of females and males represented) and *T. cruzi* infection status (approximately equal numbers of infected and non-infected specimens). Because of comparatively low sample size, only *T. gerstaeckeri* and *T. sanguisuga* individuals were included in downstream analyses, though raw sequence data for all individuals was deposited at the European Nucleotide Archive under accession number PRJEB34484 to facilitate future research. Only triatomines that had been collected alive were included in order to minimize unknown impacts on the microbiome. Upon receipt, whole triatomines were refrigerated at 4°C. After dissection, excised triatomine gut samples were kept at −20 or −80°C before DNA extraction. Associated metadata can be found in Supplementary Table 1. Triatomines were identified and dissected as previously described, including scoring the amount of blood in the gut from one to five where one is no blood in the gut and five is a large amount of fresh blood (Curtis-Robles et al., 2018b). Hindgut DNA extractions (Omega E.Z.N.A. Tissue DNA; Omega Bio-Tek, Norcross, GA, United States), *T. cruzi* infection status, and individual *T. cruzi* DTU determination testing were performed as previously described (Curtis-Robles et al., 2018a).

### *Triatoma* Gut Microbiome DNA Sequencing

PCR targeting the V4 region of the 16S rRNA gene was performed on each sample in triplicate using the 515F (5′-GTGYCAGCMGCCGCGGTAA-3′) and 806R (5′-GGACTACNVGGGTWTCTAAT-3′) primer set. All samples were prepared for sequencing using the 16S rRNA gene Metagenomic Sequencing Library protocol for the Illumina Miseq (Illumina, San Diego, CA, United States) (Illumina, Inc., 2013) using AccuPrime High Fidelity Taq DNA Polymerase (Thermo Fisher, Waltham, MA, United States). A single positive (*Escherichia coli*) and negative (molecular biology grade water) control was processed in parallel to each extraction and PCR batch. Non-amplification status of negative controls and amplification status of positive controls were verified via gel electrophoresis but were not included in the sequencing run. The multiplexed sample pool was prepared and loaded onto an Illumina Miseq for sequencing according to manufacturer guidelines (16S Metagenomic Sequencing Library Preparation Part # 15044223 Rev. B; Illumina, San Diego, CA, United States) at the University of North Texas Health Science Center’s Tick-Borne Disease Research Laboratory using a 2 × 250 v2 chemistry.

### Computational Analysis

Primers were trimmed from raw reads using Cutadapt v. 1.16 (Martin, 2011). Trimmed reads were quality filtered, merged, and checked for chimeras using the DADA2 pipeline (Callahan et al., 2016). Finally, taxonomy was assigned to processed sequences using VSEARCH v. 2.8.1 (Rognes et al., 2016) and the EzBiocloud database as a reference (Yoon et al., 2017; Supplementary Tables 2, 3). Data analysis was primarily performed within the R version 3.5.0 environment (Team RC, 2013). Diversity analyses were performed with PhILR (Silverman et al., 2017), the adonis function within the Vegan v. 2.5–5 library (Oksanen et al., 2019), and Phyloseq (McMurdie and Holmes, 2013). Differential abundance of specific bacterial taxa between *T. cruzi* positive and *T. cruzi* negative individuals was calculated using Phylofactor (Washburne et al., 2017). Phylofactor identifies clades within a phylogenetic tree that drive observed variation between independent user-defined variable groups. A hierarchical cluster dendrogram was generated using the hclust function in R using the complete linkage method. The predictive power of sample metadata on the triatomine microbial community was estimated with a random-forest classification model using the R packages randomForest (Liaw and Wiener, 2002) and rfUtilities (Murphy et al., 2010). A maximum likelihood tree for *Salmonella* was generated using the SILVA 16S rRNA database All-Species Living Tree Project (Munoz et al., 2011), PyNAST (Caporaso et al., 2009) as implemented in QIIME v1.9 (Caporaso et al., 2010), and RAxML (Stamatakis, 2014). Scripts for all read processing, analyses, and figures can be accessed at https://github.com/aemann01/triatomines.

## Results

A total of 90 triatomine specimens were initially included, six of which were removed from the dataset due to poor 16S rRNA V4 amplification. Due to uneven sampling among triatomine species, only *T. gerstaeckeri* and *T. sanguisuga* individuals were included in downstream analyses. In addition, a single *T. gerstaeckeri* sample (Tri1676) had fewer than 500 reads post quality filtering and was also excluded leaving 74 samples. An average of 58,889 reads were generated for each sample (±14,044) of which an average of 49,150 (±14,485) were retained post quality filtering (Supplementary Table 4).

**FIGURE 1:**
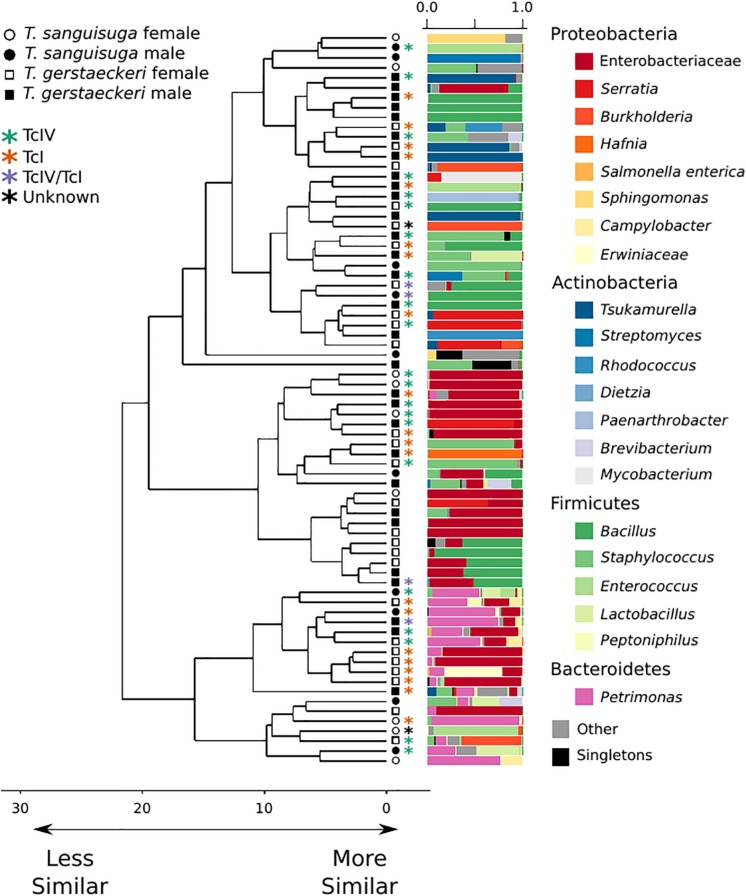
Triatomine gut microbiome diversity is loosely structured across individuals, with high interindividual variation. Hierarchical clustering dendrogram of PhILR distances. Species, sex, *T. cruzi* infection status, and DTU classification are indicated by symbols at end of dendrogram branches. Relative abundance of bacterial taxa represented in bar chart colored by bacterial taxonomic group for each sample at tips of dendrograms. Singletons (i.e., those bacterial ASVs found uniquely in a single sample) are colored in black. Axis values on dendrogram indicate the calculated distance between clusters (complete linkage).

The gut microbial community of individual triatomines was characterized by low intraindividual taxonomic diversity [average amplicon sequence variant (ASV) count = 21 ± 29; Supplementary Figure 1] and high interindividual variation that was not predicted by triatomine species, sex, collection location, blood meal score, or *T. cruzi* infection status (Figure 1 and Supplementary Figure 2). A single *T. sanguisuga* individual (Tri1117) had unexpectedly high ASV diversity with a total of 224 observed ASVs (Supplementary Figure 1). The lack of clustering of samples by *T. cruzi* infection status and species is confirmed by two alternative ordination procedures, weighted UniFrac distance and Bray Curtis dissimilarity (Supplementary Figure 3). Two measures of alpha diversity (Shannon diversity index and observed ASVs) were not significantly different between species or between *T. cruzi* positive and *T. cruzi* negative individuals (Supplementary Figure 4).

A Random Forest Classification model was used to determine the predictive power of the composition of triatomine microbial communities in accurately classifying triatomine sex, species, and *T. cruzi* infection status using 10,000 trees. Given an equal number of *T. gerstaeckeri* and *T. sanguisuga* individuals (19 randomly sampled per species), our model correctly identified *T. gerstaeckeri* in 73.68% of cases and in 57.89% of cases for *T. sanguisuga* [out of bag error (OBE): 34.21%; *p* = 0.03]. As host species is expected to impact microbial diversity, classification analyses below were performed on each species separately. Correct estimation of triatomine sex within species was not significant, with *T. gerstaeckeri* females (*n* = 26) correctly identified in 50.00% of cases and *T. gerstaeckeri* males (*n* = 26) correctly identified in 42.31% of cases (OBE: 53.85%; *p* = 0.57). While *T. sanguisuga* females (*n* = 9) were correctly identified in 77.78% of cases, *T. sanguisuga* males (*n* = 9) were correctly identified in only 55.56% of cases (OBE: 33.33%; *p* = 0.05). Finally, given an equivalent sampling of *T. cruzi*-positive and *T. cruzi*-negative individuals within species, positive *T. gerstaeckeri* were erroneously classified as negative in 55.56% of cases (OBE: 47.22%; *p* = 0.26) while positive *T. sanguisuga* were erroneously classified as negative in 33.33% of cases (OBE: 33.33%; *p* = 0.06).

A PERMANOVA was performed to determine whether bacterial diversity was predicted by triatomine species, sex, *T. cruzi* infection status, or collection location. The impact of triatomine species on microbial diversity was weak (*R*^2^ = 0.05) but statistically significant (*p* = 0.01), as was *T. cruzi* infection status (*R*^2^ = 0.04; *p* = 0.04). However, the impact of *T. cruzi* infection status was insignificant when controlling for species for both *T. gerstaeckeri* (*R*^2^ = 0.04; *p* = 0.11) and *T. sanguisuga* (*R*^2^ = 0.12; *p* = 0.11). Similarly, no significant effect was detected for triatomine collection location (*R*^2^ = 0.40; *p* = 0.20), blood meal status (*R*^2^ = 0.21; *p* = 0.20), sex (*R*^2^ = 0.03; *p* = 0.10), or DTU strain type when only TcI or TcIV positive individuals were considered (*R*^2^ = 0.14; *p* = 0.49). Instead of being predicted by the metadata collected in this study, the overall gut microbiome diversity was very loosely clustered across individual triatomine insects and tended to be dominated by bacteria in the phyla Proteobacteria, Actinobacteria, Firmicutes, or Bacteroidetes (Figure 1). The most commonly detected bacterial groups detected across triatomine individuals include Enterobacteraceae, *Bacillus*, *Petrimonas*, *Staphylococcus*, *Sediminibacterium*, and *Brevibacterium*, with 83.78% of all samples positive for Enterobacteraceae, 67.57% for *Bacillus*, and 64.86% for *Petrimonas*.

**FIGURE 2:**
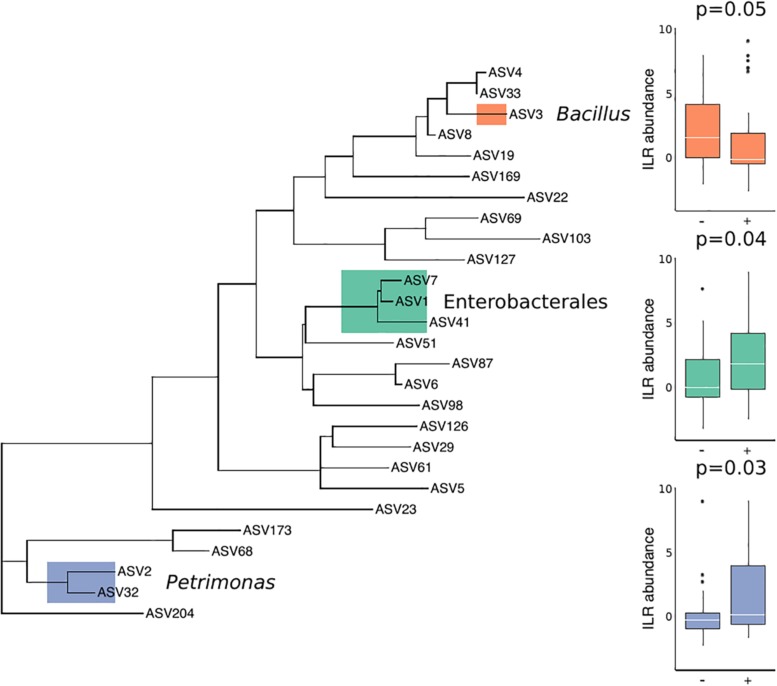
Differential abundance of three bacterial groups in *T. cruzi*-negative and *T. cruzi*-positive individuals. *T. cruzi*-positive individuals were enriched for *Petrimonas* (*p* = 0.03) and three ASVs assigned to Enterobacterales (*Serratia*, Enterobacteriaceae, and *Morganella morganii*) (*p* = 0.04) while *T. cruzi* negative individuals were enriched for a single ASV assigned to *Bacillus* (0.05). Only those ASVs found in at least 10 samples were considered for this analysis to limit the impact of rare or environmentally derived taxa.

There were no substantial differences in microbial diversity as measured by PERMANOVA, and there was low predictive power of the microbial community composition in correctly classifying *T. cruzi* positive and negative individuals, as measured by our Random Forest Classifier model. However, specific bacterial groups including *Petrimonas* (*p* = 0.03) and three ASVs in the order Enterobacterales (*p* = 0.04) were enriched in *T. cruzi* infected individuals while *Bacillus* was enriched in *T. cruzi* negative individuals (*p* = 0.05) (Figure 2).

**FIGURE 3:**
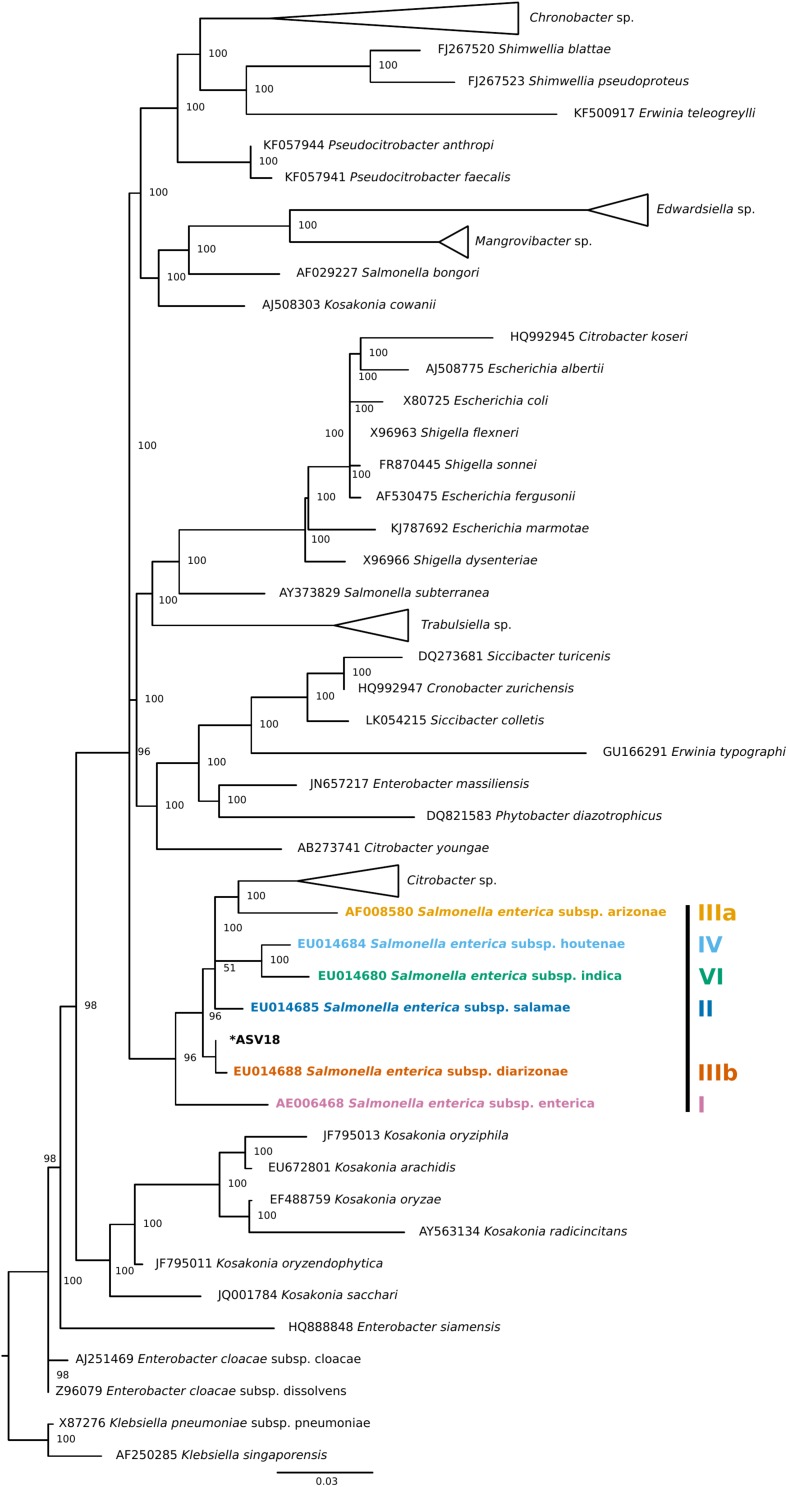
Phylogenetic placement tree of *Salmonella enterica* ASV detected in four triatomine individuals. Maximum likelihood reference tree generated by the SILVA 16S rRNA-based Living Tree Project was used as a constraint. The single *Salmonella* ASV (ASV18) was placed into the tree using PyNAST and RAxML. The closest *Salmonella* subsp. predicted by this analysis was *Salmonella enterica* subsp. *diarizonae*. When possible, genus level clades were collapsed for tree clarity.

Finally, four individuals tested positive for a single ASV that was assigned to *Salmonella enterica*. To validate this ASV, a phylogenetic tree of Enterobacteraceae 16S rRNA reference sequences generated using data from SILVA’s Living Tree Project (v 132) (Figure 3). The *Salmonella enterica* ASV detected in the current study falls most closely to the *Salmonella enterica* subspecies *diarizonae*. Importantly, all triatomine individuals that carry *S. enterica* were dissected on different days and were included in separate DNA extraction batches, reducing the chance that the detection of *S. enterica* across individuals is an artifact of cross-contamination.

## Discussion

Previous reports on the bacterial gut microbiome of triatomines have found infection with *T. cruzi* to be associated with higher bacterial diversity in the gut microbiome (Montoya-Porras et al., 2018; Rodríguez-Ruano et al., 2018). Contrary to those findings, in this study, which included a much larger number of individual samples, we found that microbial species diversity is only weakly structured by triatomine species, and is not structured by *T. cruzi* infection, sex, collection location, blood meal status, or DTU strain type. The large number of variables in this study may have masked most of the effects that *T. cruzi* exerts on its host bacterial microbiome, as reported elsewhere (Díaz et al., 2016; Rodríguez-Ruano et al., 2018). However, another study on the effect of *T. cruzi* infection status on the triatomine gut microbiome found a similar lack of impact on bacterial diversity (Waltmann et al., 2019), suggesting that increased species sampling and controls are necessary to fully appreciate the interaction between *T. cruzi* and the larger microbial community. The impact of *T. cruzi* infection on the gut microbiome of triatomines may also be related to the time since infection, which was an unknown variable in the current study due to the wild-caught nature of the triatomines. Overall, the predictive power of sample metadata for classifying the gut microbial community composition was weak. Instead, we found that the composition of the triatomine gut microbiome may reflect a mixture of true gut residents and environmental or transient microbes. Transient microbes may be acquired by triatomine insects from their immediate environment or from the blood meal host itself. As triatomine insects can survive for months without feeding (Klotz et al., 2014), the impact of microbes originating from the blood meal may be unevenly distributed or the growth of native gut bacteria may be restricted. Recent research on South American triatomines suggests that the source of the blood meal and not the amount of blood itself affects the gut microbiome of triatomine insects (Dumonteil et al., 2018). While the feeding source of triatomines in the current study is unknown, determining the source of the blood meal in future studies may clarify the diversity described here. Alternatively, the lack of consistency of microbial taxa detected within and across triatomine individuals may indicate that the triatomine species investigated in the current study are not strongly reliant on a core of gut microbial symbionts, a pattern that has been described in other insects (e.g., caterpillars: *Lepidoptera* spp., solitary bees: *Anthophila* spp.) (Hammer et al., 2019), or that the core symbiont group is obscured by transient microbes. For example, six individuals tested positive for *Staphylococcus equorum*, one for *Staphylococcus succinus*, and one for *Staphylococcus saprophyticus*, which are known members of the mammalian skin microbiome, but are also found in various environmental sources (Nováková et al., 2006; Sousa et al., 2017). The impact of transient microbes on the interpretation of microbial diversity statistics may be especially relevant to species, like triatomines, that may have a low starting resident microbial diversity in the hindgut and rely on blood meals for nutrition. If there are microbial targets living in the hindgut of triatomines that may be effective for *T. cruzi* control, the relative impact of transient microbes on the interpretation of these communities must be verified.

Despite the potential impact of transient microbes in the interpretation of overall diversity, we found a pattern of differential abundance of specific bacterial groups in *T. cruzi*-positive and *T. cruzi*-negative individuals, one of which (i.e., members of the Porphyromonadacea family – in the current study represented by *Petrimonas*) has been previously reported as enriched in *T. cruzi*-positive members of *Triatoma infestans* collected from Peru (Waltmann et al., 2019). This result motivates further investigation of diverse species of triatomine insects to clarify the relationship between specific bacterial taxa and the *T. cruzi* parasite.

The overall bacterial microbiome composition of *Triatoma* spp. presented here has been similarly observed in other studies of triatomine hindguts, in particular, a high proportion of Enterobacteriaceae (da Mota et al., 2012; Gumiel et al., 2015; Rodríguez-Ruano et al., 2018; Waltmann et al., 2019). Additionally, we found a single ASV assigned to the genus *Arsenophonus*, members of which are reported to be obligate endosymbionts in a number of insect species, including *Triatoma* spp. (Hypša and Dale, 1997; Šorfová et al., 2008; Nováková et al., 2009), in two triatomine individuals at low frequency. The low frequency of this genus is likely due to the fact that all individuals in the current study were adults and the relative frequency of *Arsenophonus* has been shown to decrease with insect age (Rodríguez-Ruano et al., 2018), though another study did not find a substantial difference in the microbial composition across life stages (Waltmann et al., 2019). It is also possible that the low frequency of *Arsenophonus* is a reflection of the sampling methods used here. Specifically, hindguts were excised from individual triatomine insects prior to sequencing, while other studies may sample the full abdomen, excreted feces, or other triatomine tissues. We found a surprisingly high proportion of reads assigned to the genus *Petrimonas*. To our knowledge, a high relative abundance of this taxon has not previously been reported in *Triatoma* spp., though recent research on *T. infestans* in South America did report differential abundances of the Porphyromonadaceae family (Waltmann et al., 2019). Members of the *Petrimonas* genus have been isolated from environmental sources and ferment carbohydrates and organic acids (Hahnke et al., 2016). A BLAST search of the four ASVs assigned to this genus aligned most closely to uncultured bacteria in the NCBI NT database at relatively low percent identity (94.86–98.02%), indicating that these might represent a novel *Petrimonas* species associated with triatomine insects.

We detected *Salmonella enterica* in three triatomine individuals, likely subspecies *diarizonae*, though our ability to resolve a particular amplicon to the subspecies level is limited by the 16S rRNA metabarcoding approach taken here. *S. enterica* subsp. *diarizonae* is commonly found in reptiles (Schröter et al., 2004) and domesticated animals (Bonke et al., 2012), and can cause salmonellosis in humans. This opportunistic pathogen has, to our knowledge, not been reported in triatomine bugs and likely is a signal of their hematophagous behavior. While it is unclear if triatomines carrying *Salmonella* spp. are able to competently transmit the bacteria either through the blood meal or via their feces, they are a potential source of *Salmonella* infection to dogs and other animals that may ingest triatomines, as well as an indirect source of infection to humans interacting with domesticated or wild animals.

Results of this study illustrate the importance of cross-species comparative research and have implications for the control of *T. cruzi* infection in natural triatomine populations. Specifically, the findings highlight that patterns of microbial diversity, in the presence of *T. cruzi* and among different triatomine species, may not be applicable across the group as a whole and question the predictability of parasite infection status on the bacterial gut microbiome in natural populations of triatomine insects. Additionally, triatomines in the current study have low intraindividual ASV variation as compared to studies of wild South American triatomines (Waltmann et al., 2019), but are comparable to the low number of OTUs found in North American triatomine adults (Rodríguez-Ruano et al., 2018). Differences in intra- and interindividual taxonomic variation may be due to differences in methodology (e.g., targeting the V4 versus the V3-V4 region) or may represent true biological variation. Differentiating between these effects was beyond the scope of this study but motivates further research. As more data are generated for diverse triatomine species in the Americas, we can potentially gain a better understanding of triatomines as insect vectors of a variety of pathogens, as well as the possibility of molecular targets for Chagas disease control.

## Data Availability Statement

The datasets generated for this study can be found in the European Nucleotide Archive under accession number PRJEB34484.

## Author Contributions

RC-R, YZ, SH, and MA designed the research. EM, ST, YZ, and RC-R performed the experiments. AM, EM, YZ, RC-R, SH, and MA analyzed the data. SH and MA provided materials and resources. EM and AM wrote the manuscript text, with input from the other co-authors.

## Conflict of Interest

The authors declare that the research was conducted in the absence of any commercial or financial relationships that could be construed as a potential conflict of interest.
