# Supplementary material for: Comparison of the Bacterial Gut Microbiome of North American Triatoma spp. With and Without Trypanosoma cruzi
Source: Front Microbiol. 2020 Mar 13;11:364. doi: 10.3389/fmicb.2020.00364 (PMC7082358; doi:10.3389/fmicb.2020.00364)
Supplement: Supplementary file 1 [file Data_Sheet_1.pdf]

<u>Supplemental Figures</u>

Allison E. Mann, Elizabeth A. Mitchell, Yan Zhang, Rachel Curtis-Robles, Santosh Thapa, Sarah A. Hamer, Michael S. Allen

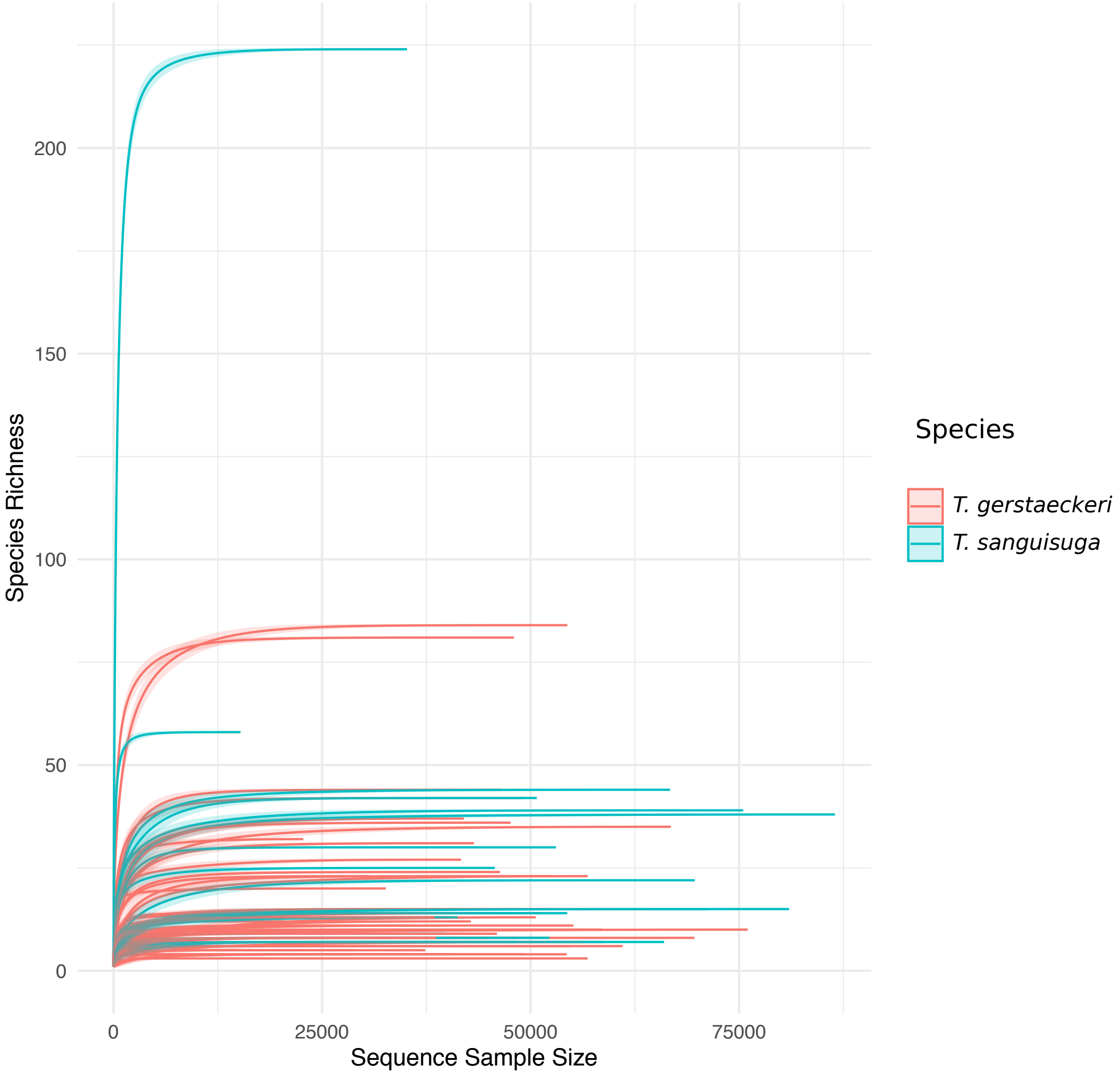

Supplemental Figure 1: Rarefaction curve analysis for all triatomine individuals. Triatomines in the current study are characterized by low diversity (fewer than 100 unique ASVs per sample) except a single *T. sanguisuga* individual (Tri1117), which had unexpectedly high ASV diversity. Lines are colored by triatomine species.

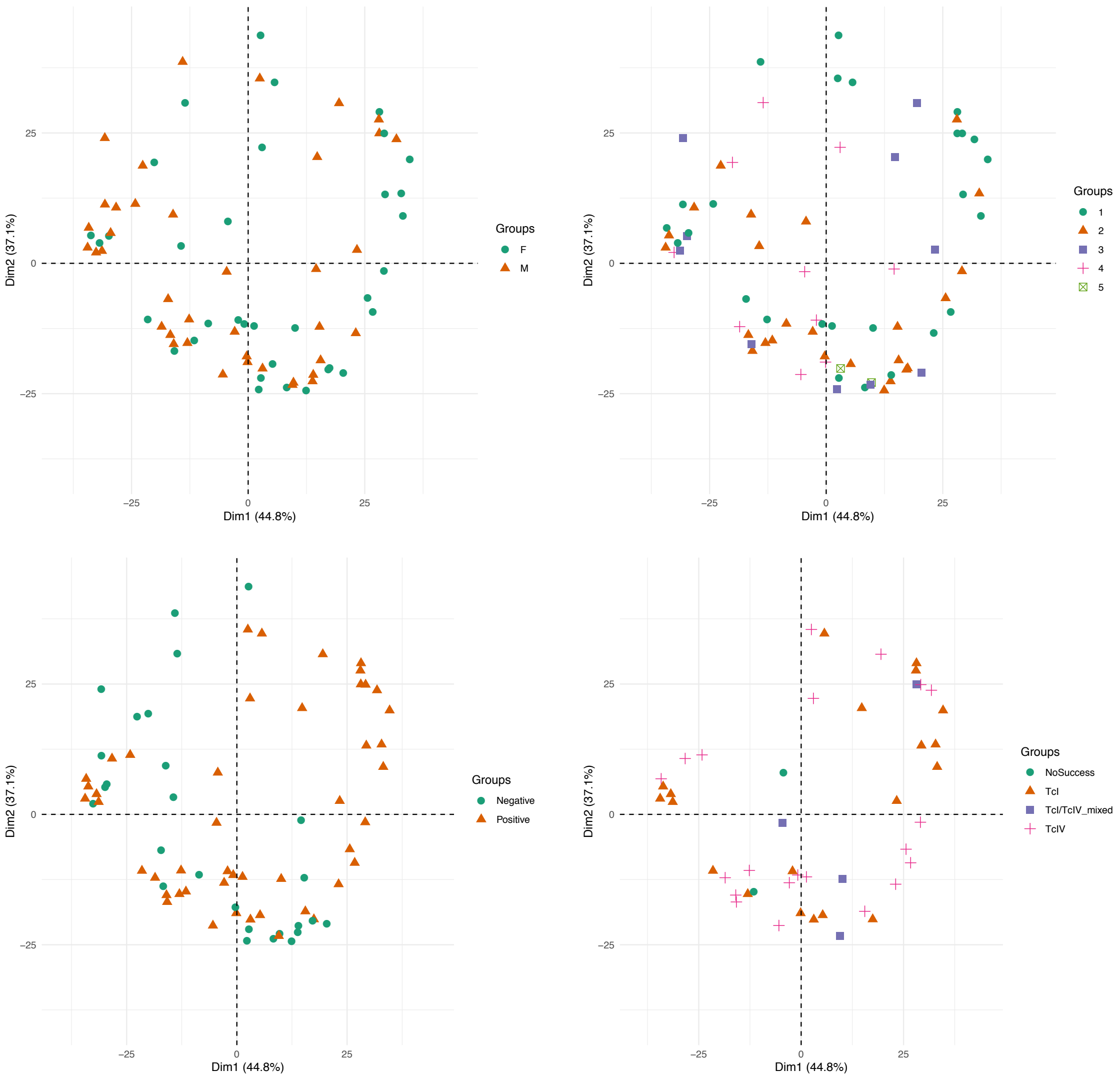

Supplemental Figure 2: Distance matrix ordination does not reveal clusters of samples by metadata categories. (a) PCA plot of PhILR transformed data grouped by triatomine sex. (b) PCA plot of PhILR transformed data grouped by blood meal score. (c) PCA plot of PhILR transformed data grouped by *T. cruzi* infection status. (d) PCA plot of PhILR transformed data grouped by *T. cruzi* DTU type. The amount of variation within the dataset is high with 44.8% explained by dimension one and 37.1% by dimension two in the above plot. The high amount of variation explained by each coordinate illustrates the high diversity among individuals in the current data set.

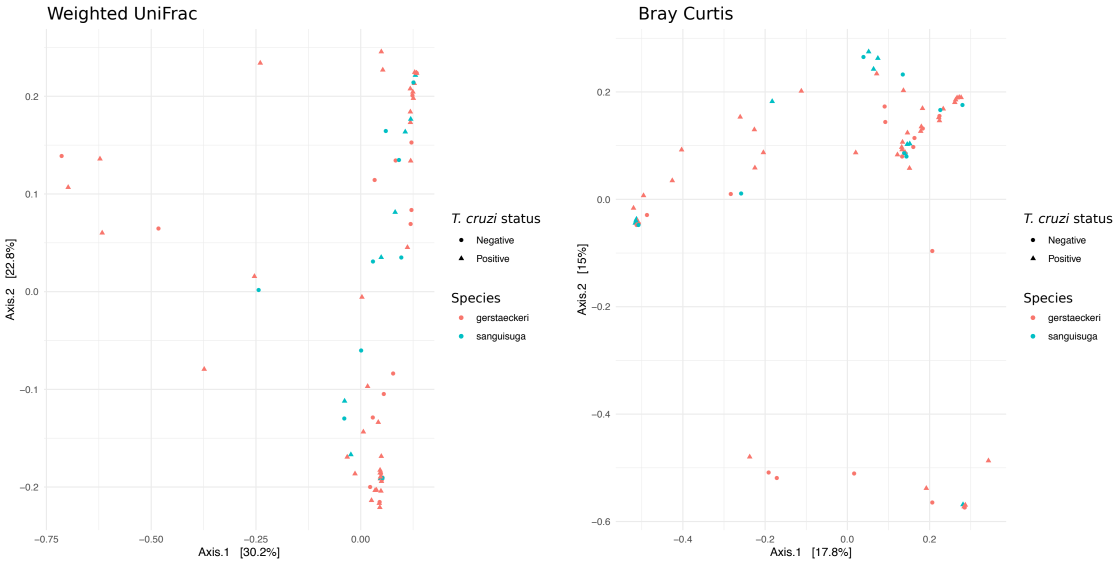

Supplemental Figure 3: Microbial community composition does not cluster by species or *T. cruzi* infection status using two alternative ordination procedures. (a) Weighted UniFrac distance. (b) Bray Curtis dissimilarity matrix. Colors represent triatomine species, shape is *T. cruzi* infection status. Data were first rarefied to 15,000 sequences per sample prior to analysis.

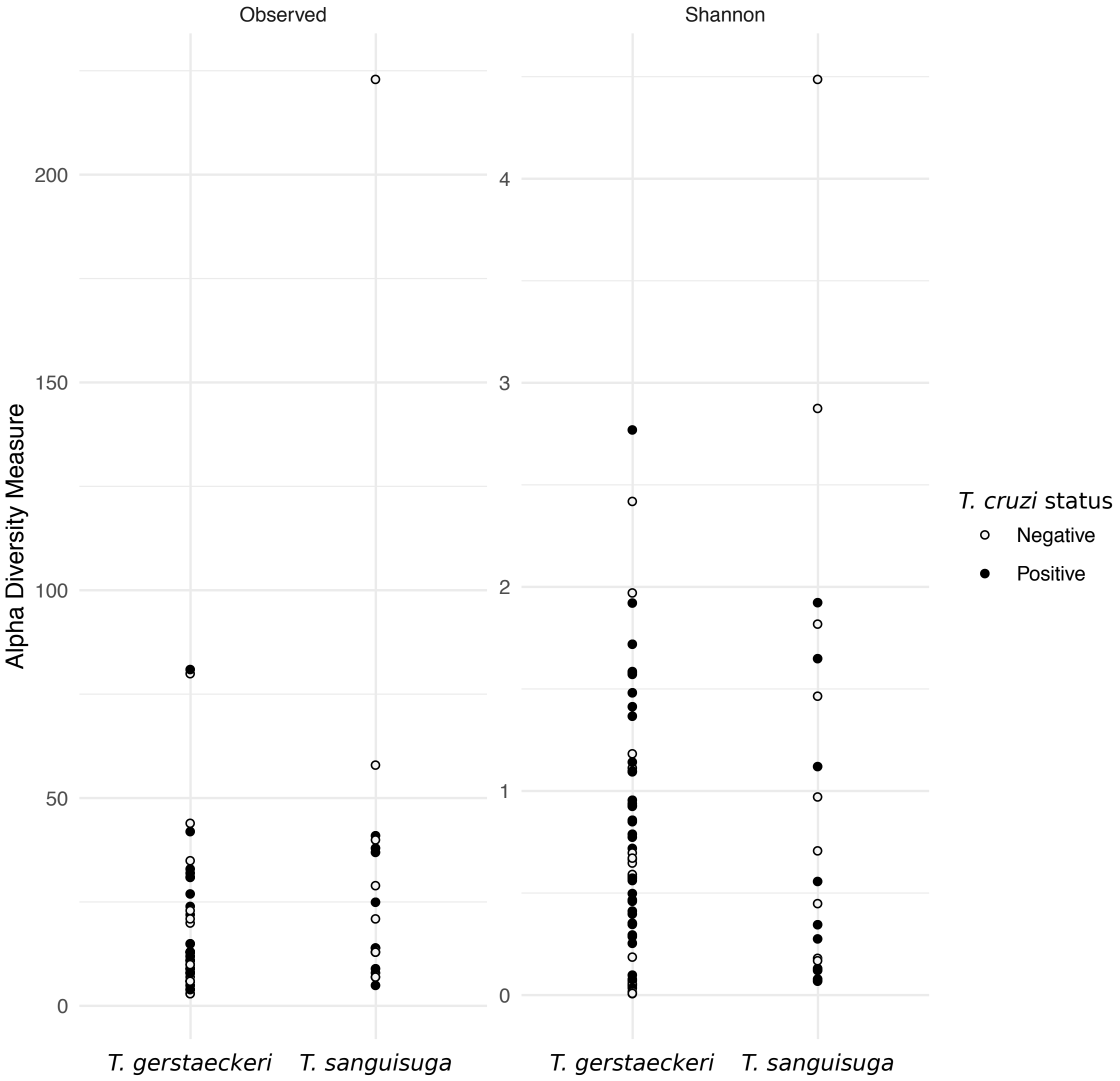

Supplementary Figure 4: Alpha diversity metrics do not reveal significant differences between *Triatoma* spp. or *T. cruzi* negative or positive indivduals. The number of observed ASVs after rarefaction between *T. gerstaeckeri* and *T. sanguisuga* is significant when considering all samples using a Wilcoxon Signed Rank Test (W=346.5; p=0.03), but not if the sole *T. sanguisuga* outlier (Tri1117) is removed from analysis (W=346.5; p=0.06). Measures of Shannon diversity are not significant between the two triatomine species (W=502; p=0.80). Neither the number of observed ASVs (W=670.5; p=0.69) or Shannon diversity (W=647; p=0.89) are significant between *T. cruzi* negative and *T. cruzi* positive individuals.
